# Early Childhood Caries Is Causally Attributed to Developing Psychomotor Deficiency in Pre-School Children: The Resultant Covariate and Confounder Analyses in a Longitudinal Cohort Study

**DOI:** 10.3390/ijerph19116831

**Published:** 2022-06-02

**Authors:** Chen-Yi Liang, Andy Yen-Tung Teng, Yen Chun Liu

**Affiliations:** 1 Department of Childhood Education and Nursery, Chia Nan University of Pharmacy & Science, No. 60, Sec. 1, Erren Rd., Rende Dist., Tainan City 71710, Taiwan; l10436@mail.cnu.edu.tw; 2 Center for Osteoimmunology and Biotechnology Research (COBR), School of Dentistry, College of Dental Medicine, Kaohsiung Medical University (KMU) & KMU-Hospital, Kaohsiung City 807, Taiwan; andytengyt@yahoo.com; 3 Laboratory of Molecular Microbial Immunity, Division of Periodontology, The Eastman Institute for Oral Health, School of Medicine & Dentistry, University of Rochester, Rochester, NY 14620, USA; 4 Center for Osteoimmunology and Biotechnology Research, Department of Oral Hygiene, College of Dental Medicine, Kaohsiung Medical University, No. 100, Shih-Chuan First Road, Kaohsiung City 807, Taiwan

**Keywords:** early childhood caries vs. dmft, psychomotor deficiency vs. CCDI aspects, pre-school kindergartners, causal interactions, longitudinal cohort study, confounders and co-variables

## Abstract

Background: Causality has recently been suggested to associate early childhood caries with psychomotor deficiency in preschoolers, where their causal interactions via other risk determinants remain unclear. Methods: To analyze such causality, we randomly recruited 123 three-to-six-year-old children in a three-year longitudinal study, where the caries/dmft measures, age/gender, BMI, amended comprehensive scales for psychomotor development (CCDI-aspects), parental education/vocation, and diet were collected for assessment of their inter-relationships. Subsequently, *t*-tests, multiple/linear-regressions, and R^2^-analyses were utilized to compare the differences of variables between age/gender, BMI, and dmft vs. relationships among all variables and CCDI-aspects. Results: In the regression modeling, there were significant differences between gender vs. age (*p* < 0.05; not BMI) regarding established associations between caries and CCDI manifests for psychomotor deficiency. As for diet vs. socio-economic status, there were significant differences when caries/dmft were at lower- vs. higher-scales (<4 and 6–10), associated with expressive language and comprehension-concept (*p*~0.0214–0.0417) vs. gross-motor and self-help (*p*~0.0134–0.0486), respectively. Moreover, diet vs. socio-economic-status contributed significantly different CCDI-spectra via expressive language and comprehension-concept (adjusted-R^2^~0.0220–0.2463) vs. gross-motor and self-help (adjusted-R^2^~0.0645–0.0994), respectively, when the caries detected were at lower- vs. higher-scales (<4 and 6–10), in contrast to those depicted without both SES diet variables (adjusted-R^2^~0.0641–0.0849). Conclusion: These new findings confirm that early childhood caries is causally attributed to developing psychomotor deficiency in preschoolers, whereas biological gender/age, not BMI, may act as viable confounders during interactions, in contrast to diet and socio-economic status, via differential low–high scales of caries activity with significant interference, respectively. Collectively, ECC-psychomotor interactions may underpin some distinct biologic vs. socio-mental/psyche attributes towards different determinants for vulnerable children.

## 1. Introduction

Early childhood caries (ECC) is the primary etiology of toothache and tooth loss in children, which is one of the most prevalent pediatric diseases, likely accompanied by long-term consequences into adulthood in later life [1,2,3]. According to the ‘specific dental plaques theory’, it is clear that caries is denoted by the penetration of certain oral microbiomes into the tooth’s hard tissues, leading to acute sequelae and/or irreversible decay in its structures (i.e., enamel, dentin, and pulp, etc.); when left untreated, it may otherwise result in some irreversible outcomes [1,2,3,4,5,6], including reduced or modified occlusal forces on mastication or neurologic vs. neural/brain stimulations, or it may impact onto the physical growth and intellectual development of functions, as reported in [7,8,9,10,11,12].

Childhood growth involves both physical and mental developments; thus, at the population level, using all available estimates and evidence measured, the key impact on developing ECC has been considered, generally, as biological (e.g., host/substrate, diet, microbes/biofilm, and time, [1,2,3,4,5,6]) vs. environmental/social issues, with all risks surrounding the saliva and mouth being involved [4,5,6,13,14]. Additional factors may also include the following: behavioral, circumstantial and lifestyle, medical/dental healthcare issues, etc. [15,16,17,18,19,20,21,22,23,24,25]. Modern research has also provided further evidence that the non-dental risks on caries/ECC may generally involve those with lower levels of socio-economic status (SES), vulnerable (sub)populations with disparities, and certain minority/ethnical vs. racial groups, etc. [15,19,20]. Some risk factors or predictors have been considered for developing ECC, including equity distributions in the (sub)populations studied [1,2,4,15,19,20,22]. In parallel, SES may act as a factor in biological or behavioral vs. conceptual access to oral healthcare in ECC-susceptible children [1,2,3,4,15,16,17,19,20,21,22,24], whose particular risks may be associated with specific oral biofilm-species, improper feeding and dietary behaviors, and environmental and self-image attributes, as well as some ill-defined underlying parental socio-mental/psyche distress or stresses. [2,4,23,26,27,28,29,30,31,32,33,34,35,36].

ECC may give rise to concerns regarding children’s general health, where the underlying pathways remain unclear at present. Our lab was interested in investigating whether ECC can impact the child’s general health regarding growth maturation and its relationship(s) to psychomotor development (via the Chinese Child Development Inventory (CCDI), by quotients [37,38,39]). To this end, we have recently shown that there is likely a causal correlation between active ECC (dmft > 3~8) and psychomotor deficiency (i.e., expressive language and comprehension-concept, etc.) in normal/regular classes of kindergarteners (i.e., without any developmentally delayed children involved), randomly selected from the country-wide regions via longitudinal designs for follow-up measures and statistical analyses [13,14,18]. Meanwhile, such likely causal interactions may be attributable by a content-reliant association through SES analyzed in kindergartener-family cohorts, whose influence was not only significantly associated with ECC progression (a biologic trait), but also with psychomotor deficiency (a social trait) in vulnerable children [21].

However, others in the field have shown that SES has long been considered one of the risk factors, or even a predictor, for ECC development and/or progression in children, including levels of family income, parental education and occupation, and access to medical/dental healthcare facilities, etc., all of which may be attributed to the equity issue of caries distribution in the population studied [15,16,17,19,20,22,23,24,25,32]. Interestingly, such intricate causal interactions between ECC and developing the psychomotor deficiency described above are found to be further implicated by other non-biologic risks/determinants, including SES (especially maternal educations), family-income level, and the frequency of dental visits, which have all been reported in our recent preliminary analyses [21].

Therefore, the present study was specifically and scientifically aimed at (i) confirming whether ECC is a causality accountable for the development of psychomotor deficiency in different CCDI aspects, and (ii) measuring and detecting any dominant role/contribution of the confounding variables (i.e., age, gender, BMI, SES, and diet, etc.) that may interfere with caries-psychomotor manifests in three-to-six-year-old kindergartener-family cohorts randomly recruited from the central and southern regions of Taiwan.

## 2. Method and Materials

To study the causal interactions addressed above, we have designed a three-year longitudinal study via the random recruitment of children aged three to six years old, whose baseline vs. follow-up caries activities and their growth-and-development aspects were collected via standardized protocols from randomly selected central and southern regions of Taiwan. Based on our recent reports [13,14], the protocols employed for the current study were directed via the same measures and criteria previously established [18,21], which are described below regarding their capacity to achieve the objectives stated above. After obtaining legal consent, the caries/dmft measures, as independent variables; the demographic data of age, gender, and BMI (height/weight); and the amended comprehensive scales (CCDI-aspects as the dependent variable) regarding psychomotor development, parental educations vs. occupations, and dietary information by categories were collected for the calculation of their inter-relationships. Analyses for assessing statistical significance were then utilized to (i) confirm whether ECC is causally involved with psychomotor deficiency and (ii) investigate any significant role or contribution of the potential confounders that may interfere or interact with the ECC-psychomotor axial traits in the kindergartener-family cohorts being studied.

### 2.1. Subject Selection

This current project was approved by the IRB and the ethics committee of Kaohsiung Medical University, Kaohsiung, Taiwan, (KMUHIRB-SV(1)-20180054 & -SV(1)-20210005), where informed consent was obtained after the protocol was thoroughly explained to the parents or legal guardians via calibrated interviewers. For the project’s sample size estimate, we applied the published data [40] with which our estimated sample size and the criteria selected to reach statistical significance have been described [21], allowing us to adopt the random recruitment and standardized protocols in the same way [18,21]. Based on our previous reports [18,21] and the present study design, the children recruited all came from regular/normal classes of kindergarteners in their respective programs without any developmentally delayed or disabled children; therefore, the participating three-to-six-year-old children enrolled in our present study did not employ any of the specific inclusion or exclusion criteria listed in the original approved IRB protocols. We included three-year-old kindergartens for subject recruitment and sampling (30% drop-out estimated) based on “first-come, first-serve”. Because we have reported the prior results derived from the random selection of southern Taiwan, the central region was then randomly selected for the present study from the rest of the geographic regions, including the northern, eastern, central, and mountain areas, as described earlier [18,21]. Both regions were either anticipated or already known to exhibit different levels of dmft activities [18,21,40] compared to our national average [40]. All preschool three-year-old children who were recruited and consented went through the standardized oral exams by qualified dental personnel in our team. Meanwhile, the subjects’ demographic data, including age, gender, BMI and food/diet intakes, parental education/occupation, psychomotor developments (via CCDI-aspects) and behaviors were all supplied via surveys completed by the caregivers and/or parents separately and independently, and then entered into the database for analyses. The approved procedures were performed at dual timepoints; firstly, when the children were three-years-old, after their entry into the project (at baseline) then, later, by or before the ages of four and a half to six years (at the first follow-up), sequentially. The present analyses stemmed from the prior preliminary report [21], and a total of 123 three-to-six-year-old old preschoolers were selected, who had either completed all baseline and first follow-up clinical oral exams (e.g., a mean 17 months since the baseline; see Table 1) and CCDI and parallel full-questionnaires required; otherwise, were all-existed from computations in the present analyses As a result, the participation rate was 78.6% in the central vs. 75% in southern region, whereas the averaged completion rate post all-exits was 77.4%, accordingly.

### 2.2. Oral Examinations for the dmft Measures

The oral clinical exams were all performed based on WHO guidelines (WHO 1997; oral health surveys: 4th ed.). Each participant received an oral examinations by two qualified dentists, where the resulting diagnostic reproducibility was estimated as κ = ~0.80–0.84 [18,21], based on the same WHO guidelines. The oral examinations included the number of decayed, extracted, or filled primary teeth, and the sums of all these measures were computed for the dmft scores in the datasets of the present study.

### 2.3. Body Mass Index (BMI) and Dietary Statue

BMI is a measure of weight by height, as the WHO recommends, and is used to classify weight and obesity. It is defined as the weight in kg divided by the square of the height in meters (kg/m^2^). Normal BMI for pre-school children has been defined by the Department of Health and Welfare of the Executive Yuan, Taiwan. Weights and heights of all participating children were measured using the same electronic meter. Food intake was surveyed via questionnaires completed by parents, legal guardians, or kindergarten teachers to document the children’s dietary conditions. The questionnaires were derived from the standardized questionnaire of the Nutrition and Health Survey, Taiwan (NAHSIT), which included the frequency and type of food-intake [13,14,18]. Based on all 33 items categorized in the NAHSIT, four new items were added: fried foods, high-fat snacks, high-sugar snacks, and sweetened beverages [12]. Subsequently, data from the parents’ questionnaires were pooled with those of the kindergartens regarding the subjects’ daily timing of food intake (Cronbach α = 0.854). Accordingly, eight categories were used for the assessments, including three nutrient categories (i.e., calcium, protein, and carbohydrates) and five food categories (i.e., vegetables and fruits, sweetened beverages, non-sweetened beverages, candy, and fried food) [14,18].

### 2.4. The CCDI and Assessment for Psychomotor Development and Deficiency

The Chinese Child Development Inventory (CCDI), as modified by Hsu et al. [25,37] and derived from the original Minnesota Child Development Inventory (MCDI), 1978 [37,38,39], has been frequently employed to assess children’s and infants’ physical and mental developments for general health and healthcare assessment [14,18,39,40]. Comparable to the original version, the aspect scales of the CCDI included a total of 320 items over seven developmental aspects (i.e., gross motor, fine motor, expressive language, comprehension-concept, situation comprehension, self-help, personal–social) and one overall summary scale (as the general development scale). Therefore, it is suitable for infants of six months up to six-year-old children and includes a comprehensive questionnaire completed by the caregiver and/or healthcare professionals. In addition, the validity of the CCDI has been adequately appraised; its correlation coefficients for all developmental areas analyzed have been above 0.90, and its general development area is ~0.837 [14,18,38,39]. Furthermore, the reliability of the CCDI in all test aspects has been estimated at ~0.934, and the mean score of differences between the pre-test vs. the post-test is approximately ~1.61% (data not shown). Despite that, it has been estimated that the CCDI correlation to that of the Draw a Person (DAP) test is as good as 0.70, and that of the Chinese version of the Denver Developmental Screening Test is higher than 0.70 [14,18,38,39].

### 2.5. Acquisition of the SES Information and Data

Acquisition of the family and personal information regarding children’s paternal vs. maternal education/occupation was derived from the surveys via questionnaires completed by the parents and/or legal guardian to report the subjects’ family status, based on the approved IRB protocols along with the standardized Health Surveys of Taiwan [18,41]. Meanwhile, general information vs. statistics regarding family income levels in the town, city, and region and the national GDP level were obtained from the database of the Ministry of Internal Affairs. All of the healthcare-related documentation for information, including the frequency of accessing national health insurance programs for medical, dental, and drug/prescription services on the registrations and their usages for the applied statistics were obtained from the official registry of the Ministry of Health and Welfares of Taiwan, as listed and publicized, respectively, at the websites http://ebas1.ebas.gov.tw/pxweb/Dialog/statfile9.asp (accessed on 22 June 2021) and https://www.stat.gov.tw/ct.asp?xItem=968&ctNode=513 (accessed on 22 June 2021). Additionally, the parental educational/occupational classes employed for the present SES documents were derived from the official records of the Ministry of Internal Affairs of Taiwan [42,43].

It is noted that the analytic complexity of SES tools and its contents cannot be over-simplified, as SES varies from one neighborhood/region/country to another. Therefore, in order to empower the statistical methods employed for data analyses and comparisons, we have complied and recorded the resultant parental education/occupations from the questionnaires in the official surveys collected, and the family/household income levels vs. dental visits and experience from the regional datasets vs. the national healthcare insurance program of the governmental registry, respectively, to represent the content gradients of SES in the present analyses.

### 2.6. The Statistical Analyses

In the present study, all datasets employed were analyzed using the IBM computing software SPSS-Statistics (SPSS 22, IBM Corp., Amonk, NY, USA). The level of statistical significance was set at *p* < 0.05; students *t*-test was applied to test and compare the variables, including caries (dmft) and the measures of CCDI aspects among all subjects from baseline and first follow-up of the central vs. the south regions. Subsequently, a multiple regression model was employed to analyze the relationships between all variables addressed in the study. The R^2^ coefficient of determination (R-squared [44]) and the analysis of correlations in a longitudinal cohort setting were applied to test, evaluate, and model the interactive differences (0~1) between the key variables under investigation for attributable explanations, indicating the analytics applied to resolve how much a difference in one variable (i.e., BMI, SES, diet) can be accounted for by a difference in other variable(s). Thereby, one-way ANOVA, multiple/linear-regression, and R-squared analyses were used to compare the differences in variables between age, gender, BMI, and dmft vs. the relationships between all covariables and CCDI-aspects for their comparable significance.

## 3. Results

A total of 123 kindergarteners recruited from the central (*n* = 95; participation rate 78.6%) and south (*n* = 64; participation rate 75%) regions of Taiwan, out of an original 169 children, were enrolled in the present longitudinal cohorts, based on our recent preliminary reported analyses [21]. Participants had to have completed the clinical oral exams and all questions in the questionnaires/surveys at both baseline and first follow-up before being entered into the current datasets (i.e., 65 males: 52.8%; 58 females: 47.2%; see Table 1), unless information already existed. Meanwhile, for the age, gender, CCDI-aspects, dietary items (such as frequency/day), and BMI measures (e.g., height/cm vs. weight/kg) were all listed for analyses (see Table 1), while, information regarding SES (i.e., parental education/occupation, family income levels, etc.) was sub-categorically entered in Table 1, Table 2, Table 3 and Table 4 shown below for computations and statistics. Importantly, there was a significant difference in the ECC/caries activities detected over time, by dmft measures, between the baseline (at mean-age: 3.23 ± 0.60 yrs.; scored 3.92 ± 3.39) and first follow-up (at mean-age: 4.66 ± 0.60 yrs.; scored: 8.56 ± 3.65), whose time-interval was, on average, 17 months (*p* < 0.0001; see Table 1). When efforts were made to analyze potentially qualifiable and/or quantifiable differences in different dmft scores (i.e., 0–10 scales, as independent sole-variable) and individual CCDI-aspects, there were significantly different variations detected over the scattered CCDI manifests between baseline (i.e., dmft in 4–8 scales: general development scale) and first follow-up measures (i.e., dmft in 3–4 vs. 9–10 dual scales: comprehension-concept and self-help, etc.), while they were in contrast to those dmft scales analyzed with the included controlled variables, including gender, age, and “gender—age” for statistical comparisons (see Table 2). Furthermore, some comparable results were seen when employing adjusted R-squared analyses, where significant attributions via various CCDI-aspects for accountable differences detected in separate groups (such as dmft (in scales), dmft—gender, dmft—age, and dmft—(gender-plus-age)), yielded through vastly different dmft scales and resultant CCDI manifests, spread out in a non-overlapping manner without common attributed domains (see Table 2). Therefore, such results prompted us to consider and implement gender and age as the controlled variables, while contemplating potential confounding factors for proper analyses in our current model.

Moreover, we analyzed the potential influence and/or interference of BMI, diet, and SES sub-categories for their individual contribution to ECC-psychomotor interactions through different CCDI manifests detected in the baseline (mean dmft = 3.92) and first follow-up measures (mean dmft = 8.56), while dmft (in 0~10 scales) was coupled with “gender—age” as multiple independent variables (i.e., IVs) for statistical and R-squared analyses (Table 3). The resulting analyses showed that BMI was not correlated with being a sole-confounder per se, because there was no significantly attributed CCDI manifest(s) detected, as depicted in Table 3. Interestingly, there were significantly detectable differences when (i) IVs vs. IVs and diets were statistically and R-squared analyzed, showing reciprocal negative- vs. positive-interference with variable CCDI manifests detected and explainable attributes within baseline and first follow-up measures, and (ii) IVs vs. IVs and SES were statistically and R-squared analyzed, resulting mostly in all-positive interferences to the signified CCDI manifests detected, with accountable attributes among all first follow-up measures (Table 3). However, regarding the R-squared analyses for comparing IVs—diets and IVs—SES groups, there were no consistently and significantly explainable attributes based on the resulting CCDI manifests that could differentiate such diets from SES in general (see Table 3). Nevertheless, it is conceivable that diets vs. SES each behaved or acted differently on the CCDI manifests when the caries profile was lower (i.e., 0–4 scales, referring to expressive language and comprehension-concept, etc.; see IVs—diets in first follow-up of Table 3), in contrast to those of higher dmft measures (i.e., 5–10 scales, referring to gross motor and self-help, etc.; see IVs—SES in first follow-up of Table 3). These findings clearly suggest that diets and SES, not BMI, are both capable of behaving as independent confounding factor(s) during ECC-psychomotor interactions in the kindergartener-family cohorts.

Subsequently, regression modeling and R-squared analyses were employed to address the relationship(s) between CCDI-aspects, as dependent variables, and caries/dmft activities, being the independent variable vs. all other independent variables (e.g., gender, age, food/dietary items, and SES sub-categories) over time in the study cohorts. The results in Table 4 show that (i) there was no significant difference ever detected for the preschoolers in the baseline phase (data not shown here), because their mean age (~3.23-yr) and caries experience (dmft~3.92) were either smaller or lower in the beginning, and (ii) on the contrary, in the first follow-up phase (on average, 17 months later), there were variably specific CCDI manifests yielding a statistical significances detected with caries activities (i.e., dmft scales; *p* < 0.05 in Table 4) and all other variables analyzed in the R-squared sectors, including age and certain SES sub-categories (i.e., mostly via maternal educations and paternal education/occupation, etc.; see Table 4).

Interestingly, expressive language vs. comprehension-concept were rather significantly and differentially associated with age and maternal education, with or without the paternal education/occupation factor, when caries activity was lower (dmft 0–2 scales) vs. higher (dmft 0–4 scales), respectively, in the first follow-up period, which were in accordance with differential attribute(s) of the accounted CCDI manifests via R-squared analyses (i.e., range 26.6–32.5%; see Table 4). Furthermore, fine-/gross-motor vs. self-help as the attributable CCDI manifests were only significantly associated with paternal occupations vs. age and maternal educations over time, while caries activity became much higher (refer to dmft 8–10 scales in Table 4 and dmft 5–8 scales in Table 3), without obvious changes in any of their accountable CCDI manifests for psychomotor deficiency.

## 4. Discussion

Children’ growth and development involve both physical and mental/psyche aspects, whose interactions are essential for achieving general good health and wellbeing. ECC, if not treated, can progress into a prevalent and costly disease that impacts later life. Recent research development on ECC has highlighted additional non-dental risks other than the classical 4-theme cycles [1,2,3,4,5,6,13], such as lower levels of family-income, parental background, especially regarding maternal education, and certain vulnerable groups associated with the disparities or background of ethnical/racial minorities, etc. [14,15,16,17,19,20,21,22,23,24,25,26,27,28,29,30,31,32,33,34,35,36]. Therefore, it is critical to identify and understand these additional and/or unforeseeable factors in high-risk sub-populations for ECC, which will bring forward the development of effective protocols for proper treatment and management for its prevention. Eventually, through such efforts, children’s oral health can be improved, and subsequent financial and social costs can be further reduced for overall improvements.

We first explained that ECC (dmft > 3~8) may be positively associated with developing psychomotor deficiency (i.e., comprehension-concept, expressive language, etc.) via a bi-township cross-sectional design in four-to-six-year-old children [13,14,18]. Later, the preliminary analyses of our recently launched three-year cohort study using the same randomization and standardized recruitment protocols in three-to-six-year-old kindergartener-family cohorts suggested that (i) there was a likely causal relationship between ECC and developing psychomotor deficiency in growing children and (ii) there were unforeseeably accountable attributes of SES (especially maternal educations) strongly associated with certain CCDI manifests (i.e., gross motor and personal–social) and caries activity measured over time, via multivariate and R-squared analyses [21]. Herein, the analytic results of the present longitudinal cohorts based on the same recruited normal/regular-classes kindergartener-family cohorts (*n* = 123; without any developmental delayed or disabled children), that were twice-measured and analyzed over time, provide unequivocal evidence and confirm that ECC/caries does indeed causally account for developing psychomotor deficiency in young kindergarteners. In addition, such significantly different dmft scores detected between baseline and first follow-up (Table 1) are consistent with the higher vs. lower caries dichotomy seen in our prior bi-township cross-sectional study, both of which established the interactive correlations revealed in our model, as reported and described in [18].

Notably, it is worth-mentioning that, in particular (see Table 3 and Table 4), the sub-categories of SES (especially maternal educations), dietary items (i.e., vegetable and fruit, sweets, etc.; data not shown here), and age itself have manifested significantly more attributable influences onto the ECC-psychomotor/CCDI causal interactions encountered in the present kindergartener-family cohorts (i.e., lower-scale vs. higher-scale caries/dmft profiles; Table 3 and Table 4) than those that caries/dmft activities did alone [1,2,3,4,5,6,13]. Importantly, gender and age, known as the natural vs. biological bound characteristics, and identities and/or personalities, carry pervasive differences through our lifetime. These naturally bound characteristics and/or identities, with few exceptions, are generally viewed as difficult to change or modify [44,45,46], yet the dietary items vs. SES sub-categories (as independent variables) may fall into the biologic vs. social/mental associations, respectively, during their causal interactions with the ECC-psychomotor deficiency axis (or trait), provided that the scientific interpretations or justifications are properly rationalized and integrated [21,30,31,33].

The resultant statistical, multiple regressions and R-squared analyses from baseline and follow-up measures (Table 4) are in high concordance with the previous findings that ECC was likely causally involved in the development of psychomotor deficiency, whose courses of progression via low vs. high caries cascades onto each prospective CCDI manifest and may be linked with other specific confounders (i.e., gender, age, SES, and diet, etc.; [18,21]), and not BMI, through different levels or magnitudes of interferences. In addition, such results, especially relating to maternal education, were also found to be significantly associated with the gross motor vs. personal–social manifests in our prior report (Table 2 of [21]), emphasizing the critical maternal influence under the content-gradients of SES categories in such ECC-psychomotor drives for the oral health conditions engaged [18,21,26,32,34,36]. Conversely, the limitations of the present analyses include the following: (i) despite the computational and analytic statistics made to analyze the roles of confounders or interferences (i.e., age, gender, BMI, diets, SES sub-categorical variables, etc.; Table 2, Table 3 and Table 4), a clear roadmap to the ECC-psychomotor axis is still lacking, and (ii) further to this missing roadmap, the step-wise impact of SES-psychomotor trait(s) over ECC-psychomotor causal interactions may not be properly interpreted at present.

Moreover, this intriguingly novel finding that SES (i.e., maternal and paternal backgrounds, etc.) and/or SES vs. diet generally contribute more to the ECC-psychomotor deficiency axis than onto traditional ECC activity may enable bridging the biologic vs. social interactions on the existing risks for ECC, thereby signifying the development of psychomotor deficiency over time [21]. For example, it has recently been shown that there were good correlations between: (i) a home-parenting environment and cognitive and psychomotor development for children under five years old, and (ii) preschool children diagnosed with attention deficit hyperactivity disorder (ADHD) and the parents’ perception of difficulties in several dimensions of development and global learning difficulties, such as expressive language, comprehension, and fine motor skills, as well as in emotions, concentration, behavior, and relationships [47,48]. Similarly, comparable findings may be extended to younger toddlers with developmental delays, where such delays can also affect the children’ physical, cognitive, communication/speech, social, emotional, or behavioral skills [49,50]. Though there were the same levels of categorical SES measures in our prior [21] and present cohort study, psychomotor deficiency cannot be solely attributed to the social characteristics described in the SES. Conceivably, such events may emerge at some critical stages, likely throughout childhood growth via language communications and some peer social/psycho-mental engagements [13]. Scientifically, we will need to investigate and further implement the differential roles and/or contributions of specific SES vs. dietary categorical measures regarding the hypothesized theme proposed [26,27,28,29,32,34,35,36,48], consisting of a direct neurophysiologic path and/or an indirect one external to the neurologic circus [13,18,21].

How the ECC-biologic deed is developed in growing children can be determined by biological exposures or behavioral vs. environmental patterns, whereas the natural vs. behavioral issues are affected by the onset of social circumstances [13,15,16,17,19,20,22,40]. Thus, such biologic vs. socio-mental interactions affect the dental/healthcare our children likely require, and their social environments (i.e., SES and related risks) affect the dental/healthcare they could have received [15,23,26,27,28,29,31,32,33,36,40,51]. It is prudent that different SES sub-categories and/or dietary constituents, along with their social/psyche environments (i.e., parenting influence, etc.) will have different health behavior patterns and health outcomes, whereas lower SES has been shown to be likely associated with poorer in-house diets, which potentially increases the risk for ECC and developing ECC-psychomotor deficiency [15,16,17,19,20,22,24,25]. Psychomotor abilities and learning dynamics underlie the most fundamental developments in children. Based on the present analyses and new findings obtained regarding ECC-psychomotor causal interactions in growing kindergarteners, a novel challenging hypothesis is being proposed, termed ‘the multi-directive determinants model’, which is to guide our new research, whose potential socio-mental/psyche implications on the prevention of ECC-psychomotor deficiency vs. its underpinning determinants will be important for future research.

Though the interplay and underlying mechanisms of the ECC- vs. SES/Diet-psychomotor interactions remain unclear at present, it is clear that the progression of ECC (e.g., SES and dietary issues in ECC-high regions, etc.) can cause the development of psychomotor deficiency. Thus, before the full substantiation of their sequelae and implications are further explored, it is important to understand the impact of critical interactions beyond the perspective of parent–child vs. parenting experience and family–neighborhood atmospheres (i.e., behaviors) that may modulate or jeopardize the risk determinants of the ECC- vs. SES-psychomotor traits on the child’s verbal/language skills, and complicating factors. In addition, based on our analyses, it may be surprising for parents when they start to witness certain signs/symptoms of deficiency in a child’s language or verbal skills along with social or behavioral withdrawals from peer communications, etc., when their child’s caries score is practically over 4 (i.e., ~3–8; [18,21]).

Therefore, the confirmed ECC-psychomotor causal interactions and ECC–SES (and-Diet)-psychomotor axis described herein, once fully substantiated, may become a well-perceived framework beyond that which has not been anticipated or realized in the past. By then, any critically rectified means of strengthening the important independent variables or interferences and deploying financial/fiscal implementations through the content-gradients of SES vs. dietary determinant(s) will enable us to achieve a significant improvement of ECC-psychomotor developmental traits for better oral health and related sequelae in our preschool children. Eventually, such an in-depth understanding of the key ECC-psychomotor domain traits identified will help us to develop effective anti-ECC guidelines, both for parents and for public oral healthcare programs, and global prevention strategies for the policymakers to promote the psychomotor/social wellbeing of our young children in the near future.

## 5. Conclusions

Based on both our recent reports [13,14,18,21] and on new findings, unequivocal evidence can be presented to confirm that (i) ECC is indeed causally attributed to developing psychomotor deficiency in growing preschoolers over time, and (ii) ubiquitous characteristics associated with gender and age as biologic variables, and critical independent confounders, such as the SES sub-categories (especially maternal educations) and dietary items, not BMI, may contribute differently to the variable attributes of CCDI manifests via differential lower- vs. higher-EEC/caries-directed psychomotor deficiency, whose underpinning interactions may progress through distinct biologic vs. socio-mental/psyche paths/trails to risk determinants in the vulnerable children affected. Further understanding of these intricate interactions among the critical issues, i.e., ECC-/SES-(and diet) psychomotor axial traits during a child’s development will likely help us in the long run to establish better oral healthcare programs and prevention strategies in aiding the well-being and overall fine development of young children.

## Figures and Tables

**Table 1:** Demographic measures of the baseline and first follow-up on caries (dmft), age, gender (M/F) and the CCDI-aspects, dietary items (such as frequency/day) and BMI (height and weight) by the gender counts (M/F).

	Baseline					First Follow-Up				
Caries (dmft: Mean ± SD)	3.92 ± 3.39					8.56 ± 3.65 (* *p* < 0.0001: dmft: Compared to Baseline)				
Age: Mean ± SD (Children No. of 2/3/4/5/6-yr)	3.23 ± 0.60-yrs. (11/73/39/0/0)					4.66 ± 0.60-yrs. (0/7/29/86/1)				
Gender (M/F)	M (65)		F (58)			M (65)		F (58)		
CCDI-Aspects	Mean	SD	Mean	SD	*p*-Value	Mean	SD	Mean	SD	*p*-Value
Gross motor	97.18	7.75	97.61	7.32	0.7525	96.16	4.83	94.93	5.25	0.1764
Fine motor	100.85	10.03	103.79	9.61	0.1000	101.39	6.93	101.07	5.98	0.7864
Expressive language	103.62	11.32	105.21	7.71	0.3693	99.02	6.39	98.49	4.16	0.5942
Comprehension conceptual	119.94	19.03	124.30	13.42	0.1487	113.70	10.49	113.00	7.89	0.6774
Situation comprehension	99.51	11.15	103.07	9.98	0.0656	98.00	9.12	100.44	6.98	0.1019
Self-help	94.49	12.35	98.62	11.83	0.0617	96.27	9.63	99.47	6.47	0.0314 *
Personal–social	99.62	9.65	98.61	8.33	0.5383	96.93	8.95	95.73	8.27	0.4425
General development scale	111.69	12.21	114.74	10.38	0.1401	106.89	7.12	107.72	5.04	0.4596
**Dietary items (as frequency/day)**										
Protein	1.78	0.91	1.67	1.17	0.7720	1.72	1.10	1.36	0.84	0.0865
Calcium	3.79	1.84	3.86	3.80	0.5782	4.12	2.22	3.32	1.88	0.1469
Carbohydrates	2.43	1.51	3.02	2.53	0.0754	3.19	2.05	2.58	1.52	0.1868
Vegetables and fruit	0.39	0.40	0.47	0.79	0.9671	0.38	0.34	0.35	0.28	0.0402 *
Sweetened beverages	0.11	0.16	0.18	0.09	0.0444 *	0.05	0.28	0.12	0.29	0.2171
Non-sweeten beverages	3.70	2.14	4.06	2.90	0.7426	3.87	2.24	4.03	2.45	0.9498
Candy	1.57	1.17	1.65	1.04	0.6775	1.48	0.97	1.41	0.69	0.1194
Fried foods	0.23	0.20	0.25	0.09	0.1605	0.19	0.17	0.19	0.11	0.0139 *
**Body mass index (BMI)**	15.53	1.51	15.15	1.69	0.1879	16.23	1.84	15.81	2.18	0.2525
Height (cm)	100.85	5.43	101.27	6.20	0.6918	109.18	6.29	111.08	7.52	0.1308
Weight (kg)	15.85	2.33	15.59	2.65	0.5688	19.46	3.62	19.67	4.38	0.7723

*: *p* < 0.05; Note: the mean time-interval between baseline and first follow-up was 17 months.

**Table 2:** Statistical significance detected among the caries (dmft: as independent variable), gender, age and certain CCDI-aspects.

Baseline (Caries; dmft = 3.92 ± 3.39)		dmft Measured		dmft and Gender		dmft and Age		dmft and (Gender + Age)	
	dmft in Scales	*p*-Value	Adjusted R-Squared	*p*-Value	Adjusted R-Squared	*p*-Value	Adjusted R-Squared	*p*-Value	Adjusted R-Squared
Fine motor	≦0 vs. ≧1	0.0764	0.0177	0.0942	0.0289	0.0280 *	0.0314	0.0362 *	0.0417
Situation comprehension	≦4 vs. ≧5	0.0832	0.0165	0.0819	0.0363	0.0497 *	0.0222	0.0484 *	0.0423
Personal-social	≦7 vs. ≧8	0.0526	0.0227	0.0497 *	0.0187	0.1136	0.0471	0.1079	0.0432
General development scale	≦4 vs. ≧5	0.0422 *	0.0257	0.0420 *	0.0355	0.2054	0.1465	0.2051	0.1570
General development scale	≦5 vs. ≧6	0.0370 *	0.0257	0.0461 *	0.0342	0.2879	0.1431	0.3379	0.1521
General development scale	≦7 vs. ≧8	0.0246 *	0.0331	0.0281 *	0.0410	0.1036	0.1540	0.1162	0.1631
**First Follow-Up (Caries;** **dmft = 8.56 ± 3.65)**		**dmft Measured**		**dmft and Gender**		**dmft and Age**		**dmft and (Gender + Age)**	
	**dmft in Scales**	***p*** **-Value**	**Adjusted** **R-Squared**	***p*** **-Value**	**Adjusted** **R-Squared**	***p*** **-Value**	**Adjusted** **R-Squared**	***p*** **-Value**	**Adjusted** **R-Squared**
Comprehension-concept	≦3 vs. ≧4	0.0317 *	0.0296	0.0304 *	0.0238	0.0838	0.1558	0.08341	0.1490
Self-help	≦9 vs. ≧10	0.0189 *	0.0368	0.0353 *	0.0558	0.0241 *	0.0414	0.0472 *	0.0641

The independent variable(s) tested separately included: dmft in scales vs. dmft and gender, dmft and age, and dmft and (gender and age). *: *p* <0.05.

**Table 3:** Statistical significance detected and R-squared measured among the multiple independent variables (IVs) and IVs plus BMI, diets, and SES individually with certain CCDI-aspects.

Baseline (Caries; dmft = 3.92 ± 3.39)		IVs		IVs and BMI		IVs and Diets		IVs and SES	
	dmftin Scales	*p*-Value	Adjusted R-Squared	*p*-Value	Adjusted R-Squared	*p*-Value	Adjusted R-Squared	*p*-Value	Adjusted R-Squared
Fine motor	≦0 vs. ≧1	0.0362 *	0.0417	0.0348 *	0.0445	0.2492	0.0376	0.0297 *	0.0847
Comprehension-concept	≦0 vs. ≧1	0.0706	0.0729	0.0696	0.0700	0.2672	0.1680	0.0464 *	0.1375
Situation comprehension	≦4 vs. ≧5	0.0484 *	0.0423	0.0511	0.0343	0.2350	0.1551	0.1380	0.0446
**First Follow-Up (Caries; dmft = 8.56 ± 3.65)**		**IVs**		**IVs and BMI**		**IVs and diets**		**IVs and SES**	
	**dmftin Scales**	***p*** **-Value**	**Adjusted R-Squared**	***p*** **-Value**	**Adjusted R-Squared**	***p*** **-Value**	**Adjusted R-Squared**	***p*** **-Value**	**Adjusted** **R-Squared**
Gross motor	≦5 vs. ≧6	0.0779	0.0454	0.0782	0.0375	0.1464	0.0243	0.0134 *	0.0737
Gross motor	≦6 vs. ≧7	0.0796	0.0451	0.0375	0.0787	0.1418	0.0248	0.0136 *	0.0735
Gross motor	≦7 vs. ≧8	0.1213	0.0397	0.0319	0.1202	0.1131	0.0282	0.0254 *	0.0645
Fine motor	≦9 vs. ≧10	0.1570	0.0398	0.0382	0.1492	0.0417 *	0.0525	0.1412	0.0955
Expressive language	≦0 vs. ≧1	0.1906	0.1103	0.1042	0.1776	0.0323 *	0.2463	0.1686	0.1518
Expressive language	≦1 vs. ≧2	0.3103	0.1051	0.0987	0.2940	0.0385 *	0.2440	0.2543	0.1473
Comprehension-concept	≦2 vs. ≧3	0.1921	0.1397	0.1507	0.1472	0.0258 *	0.2415	0.2722	0.2051
Comprehension-concept	≦3 vs. ≧4	0.0834	0.1490	0.1628	0.0517	0.0214 *	0.2440	0.0704	0.2197
Situation comprehension	≦2 vs. ≧3	0.2473	0.0089	0.2535	0.0006	0.0273 *	0.0220	0.4508	0.0417
Self-help	≦9 vs. ≧10	0.0472 *	0.0641	0.0409 *	0.0750	0.0180 *	0.0925	0.0486 *	0.0994
General development scale	≦6 vs. ≧7	0.0196 *	0.0849	0.6249	−0.0263	0.2635	0.0410	0.0296 *	0.0649

IVs: multiple independent variables (e.g., dmft in scales, gender, and age) included. *: *p* < 0.05.

**Table 4:** Regression model for relationships and the R-square measured between all variables and certain manifests of psychomotor deficiency.

CCDI-Aspects	dmft in Scales/*p*-Value	Other Variables with Significance	Adjusted R-Squared
Expressive language	≦0 vs. ≧1/0.0421 *	**age, maternal educations**	**0.2657**
Expressive language	≦1 vs. ≧2/0.0266 *	**age, maternal educations**	**0.2720**
Comprehension-concept	≦0 vs. ≧1/0.0355 *	**age, paternal educations and occupations, maternal educations**	**0.3236**
Comprehension-concept	≦1 vs. ≧2/0.0361 *	**age, paternal educations and occupations, maternal educations**	**0.3234**
Comprehension-concept	≦2 vs. ≧3/0.0326 *	**age, paternal educations and occupations, maternal educations**	**0.3246**
Comprehension-concept	≦3 vs. ≧4/0.0173 *	**age, paternal educations and occupations, maternal educations**	**0.3237**
Fine motor	≦8 vs. ≧9/0.0371 *	**paternal occupations**	**0.1101**
Self-help	≦8 vs. ≧9/0.0401 *	**age, maternal educations**	**0.1488**
Self-help	≦9 vs. ≧10/0.0160 *	**age, maternal educations**	**0.1634**

Note: In the baseline measures, there was no significance detected between all variables and manifests of CCDI-aspects; thus, not listed above. *: *p* < 0.05.

## Data Availability

The data employed in the present report is available upon email request to the correspondence and first authors, as listed herein (graceliuyc@gmail.com, l10436@ mail.cnu.edu.tw and andytengyt@yahoo.com).
